# Ex vivo comparison of drilling techniques for optimizing primary stability of zirconia dental implants in different bone densities

**DOI:** 10.1186/s40729-025-00603-z

**Published:** 2025-04-02

**Authors:** Kawe Sagheb, Senem Yildirimturk, Sebahat Kaya, Shengchi Fan, Marius Morlock, Keyvan Sagheb

**Affiliations:** 1 Department of Prosthetic Dentistry, University Medical Centre, Augustusplatz 2, 55131 Mainz, Germany; 2 Department of Oral and Maxillofacial Surgery, Istanbul University Faculty of Dentistry, Prof. Dr. Cavit Orhan Tutengil Sk. No:4 Vezneciler/Fatih, Istanbul, 34116 Turkey; 3 Department of Oral and Maxillofacial Surgery, Plastic Surgery, University Medical Centre, Johannes Gutenberg - University, Augustusplatz 2, 55131 Mainz, Germany; 4 Oral Surgery and Implantology, Faculty of Medicine and Health Sciences, University of Barcelona, Barcelona, 08907 Spain; 5 Department of Periodontology and Operative Dentistry, University Medical Center of the Johannes Gutenberg University Mainz, Augustusplatz 2, 55131 Mainz, Germany

**Keywords:** Zirconium, Dental implant, Insertion torque, Osseointegration, Primary stability, Bone density

## Abstract

**Purpose:**

The objective of this study was to investigate the primary stability of zirconia implants using varying drilling protocols, with a focus on the impact of thread cutting on insertion torque in both mixed (D2/D3) and soft (D4) bone types. The study aimed to evaluate whether reducing thread cutting could increase insertion torque and consequently improve primary stability.

**Methods:**

Four drilling protocols were developed, each varying in the degree of thread cutting: no thread cut, one-third thread cut, two-thirds thread cut, and full thread cut. Implants were placed into fresh porcine hip and tibia bones simulating D2/D3 and D4 bone. The protocols followed each implant system’s manufacturer recommendations. Insertion torque was measured using a torque indicator, and statistical analysis was conducted with the Mann-Whitney U test, with *p* < 0.05 considered statistically significant.

**Results:**

Significant differences in primary stability were observed between implant systems and drilling protocols, particularly in D4 bone. Three of the four implant systems showed improved primary stability in D4 bone when the thread cut was reduced (*p* < 0.05). One system achieved the recommended insertion torque with a full thread cut. In contrast, in D2/D3 bone, all implant systems required a full thread cut to remain within the manufacturer’s torque guidelines.

**Conclusions:**

Zirconia implant systems exhibit substantial variability in primary stability based on the selected drilling protocol and bone quality. Reducing thread cutting demonstrated improved stability in soft bone. However, excessive torque should be avoided to prevent mechanical failure, especially in systems with lower fracture resistance.

**Graphical Abstract:**

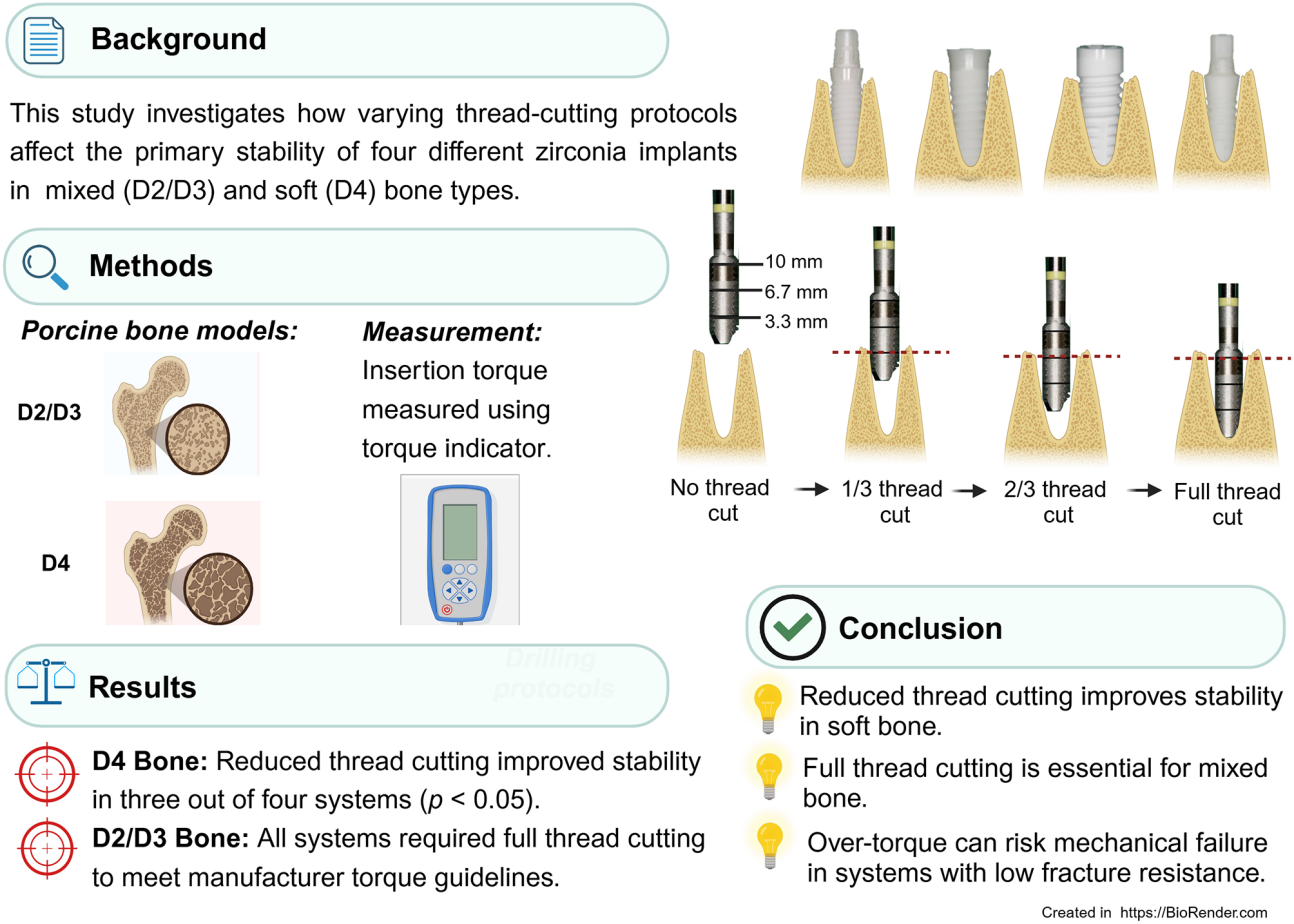

## Background

Zirconia implants have become an increasingly popular alternative to titanium implants in modern dentistry, offering a viable solution for patients with specific medical or aesthetic concerns. These ceramic-based implants are often favored for their excellent biocompatibility, resistance to corrosion, and enhanced esthetics, particularly in areas with thin gingival biotypes. Despite these advantages, zirconia implants also present distinct challenges, particularly with regard to their mechanical properties [1, 2].

One of the most critical aspects of implant success is primary stability, which refers to the mechanical stability of the implant immediately after placement. Achieving high primary stability is essential for ensuring that the implant remains immobile during the early stages of healing, thereby promoting successful osseointegration. Primary stability is influenced by various factors, including implant design, surface treatment, and, most importantly, the quality of the bone at the implant site [3, 4].

Bone quality plays a significant role in determining the mechanical engagement between the implant and the surrounding bone tissue. D4 bone, which is characterized by low density and high cancellous content, is particularly challenging for achieving primary stability. This bone type is commonly found in the posterior maxilla and presents a high risk of micromovement during the early healing period, which can compromise the osseointegration process [5]. In contrast, D2/D3 bone, which has a more favorable combination of cortical and cancellous structures, provides better mechanical support and generally allows for higher primary stability [6]. Understanding how to optimize implant placement in these varying bone conditions is essential for improving clinical outcomes, particularly in challenging cases involving soft bone.

The surgical technique used during implant placement is one of the most modifiable factors that can influence primary stability. Specifically, the drilling protocol—which determines the size and shape of the osteotomy into which the implant is placed—can significantly affect how well the implant engages with the bone [7]. In standard protocols, a full thread cut is typically performed, creating a prepared site that closely matches the thread pattern of the implant [8]. However, modifications to the thread cut, such as reducing its depth, have been proposed as a method for increasing primary stability, especially in soft bone types like D4 [9]. By reducing the depth of the thread cut, the implant may be inserted into an undersized osteotomy, which could increase the mechanical interlock between the implant and the surrounding bone. This approach has been suggested to enhance the frictional forces and mechanical engagement, leading to higher insertion torque, which is commonly used as an indicator of primary stability [8, 10].

While the potential benefits of reduced thread cutting are promising, they must be balanced against the mechanical limitations of zirconia implants. Zirconia, while highly biocompatible and esthetically superior, is more brittle than titanium and has a lower fracture toughness [11, 12]. Excessive insertion torque could place undue stress on the implant, increasing the risk of mechanical failure. Therefore, it is crucial to investigate whether modifications to the drilling protocol, such as reduced thread cutting, can enhance primary stability without exceeding the mechanical limits of zirconia implants. The null hypothesis for this study is that there is no improvement in the primary stability of a zirconia implant, measured by insertion torque, in soft D4 bone with a reduced thread cut compared to a full thread cut. This hypothesis will be tested to determine whether drilling protocol modifications provide measurable benefits in terms of primary stability.

## Methods

### Implant systems

This study evaluated four different zirconia implant systems that are commercially available. The selected systems included the Straumann PURE^®^ Ceramic Implant (Straumann Holding AG, Basel, Switzerland), Ceramic.implant^®^ (Vitaclinical, Bad Säckingen, Germany), Zeramex^®^ XT (Dentalpoint AG, Zurich, Switzerland), and Ceralog Monobloc M10^®^ (Camlog Biotechnologies AG, Basel, Switzerland). Among these, the Ceramic implant system was tested in two different diameters: 4 mm and 5 mm. This was done to assess the influence of implant diameter on primary stability, in addition to the effect of the varying drilling protocols. Therefore, while the study includes data for five implant configurations, they are derived from four distinct implant systems. Each system is composed of yttrium-reinforced zirconium dioxide and features unique surface treatments designed to enhance osseointegration. Detailed specifications, including the recommended insertion torque and implant dimensions, are presented in Table 1.

**Table 1:** Characteristics of zirconia implant systems: material composition, surface treatments and recommended torque values

Implant System	Material	Surface Treatment	Recommended Torque
PURE^®^ Ceramic Implant (Straumann)	Yttrium-reinforced zirconium dioxide	ZLA^®^ surface	35 Ncm
Ceramic.implant^®^ (vitaclinical)	Yttrium-reinforced zirconium dioxide	Sandblasted and etched	25–35 Ncm
Zeramex^®^ XT (Dentalpoint AG)	ZrO_2_-ATZ-HIP	Hydrophilic surface	20–45 Ncm
Ceralog Monobloc^®^ M10 (Camlog)	Yttrium-reinforced zirconium dioxide	CIM process	35 Ncm

Overview of the zirconia implant systems used in the study, detailing their material composition, surface treatments, and the manufacturer-recommended torque ranges for optimal implant stability. (ZLA: zirconia Large-grit Acid-etched, ZrO_2_: zirconium dioxide, ATZ: Aluminia-thoughened-Zirconia, HIP: hot isostatic postcompaction, CIM: ceramic injection Moulding)

### Bone specimens

Bone samples were obtained from fresh ex vivo porcine bone blocks, purchased from a local butcher, which serve as close analogs to human bone conditions. Soft D4 bone, predominantly cancellous, was harvested from the tibial head, while mixed D2/D3 bone, composed of both cancellous and cortical components, was taken from the pelvis. All soft tissue remnants were removed from the bone blocks, which were then cut to size using a diamond-coated cutting disc. Insertion sites were marked, and each implant system followed one of four different drilling protocols to prepare the implant bed.

### Drilling protocols and implant insertion

The four drilling protocols were designed to evaluate the effect of thread cutting on insertion torque and primary stability. The first protocol, B1, involved using a pilot drill to a depth of 10 mm without any thread cutting. In the second protocol, B2, thread cutting was performed up to 3.3 mm after the initial pilot drilling, providing partial thread engagement while leaving part of the implant bed unthreaded. The third protocol, B3, involved deeper thread cutting up to 6.7 mm, while the fourth protocol, B4, applied a full thread cut to the entire 10 mm depth. These protocols are outlined in Table 2.

**Table 2:** Drilling protocols

Protocol	Description
B1	Ball pilot drill to 10 mm, no thread cutting
B2	Ball pilot drill to 10 mm, thread cutting up to 3.3 mm
B3	Ball pilot drill to 10 mm, thread cutting up to 6.7 mm
B4	Ball pilot drill to 10 mm, full thread cutting to 10 mm

Description of the drilling protocols applied in the study to prepare implant sites. Each protocol varies in the extent of thread cutting performed after the initial ball pilot drilling to a depth of 10 mm. The protocols range from no thread cutting (B1) to a full thread cut along the entire 10 mm depth (B4), with intermediate cuts at 3.3 mm (B2) and 6.7 mm (B3)

**Fig. 1:**
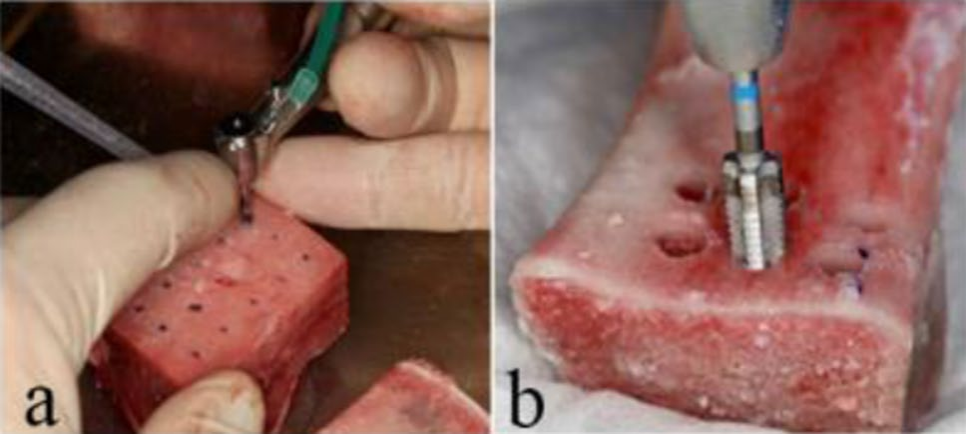
Implant bed preparation in (**a**) D4 bone and (**b**) D2/D3 bone

**Fig. 2:**
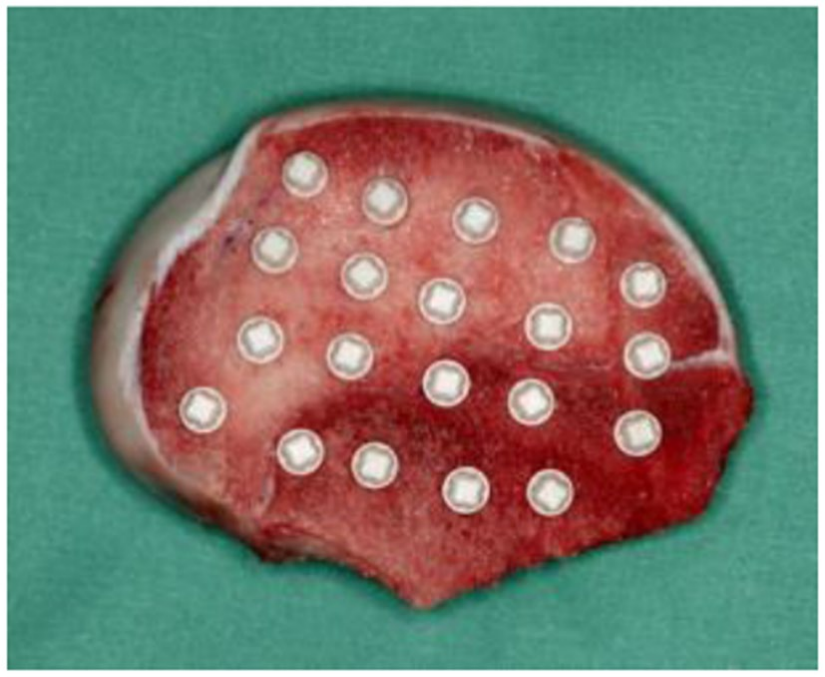
Post-insertion positioning of the 5.0 mm x 10 mm Ceramic.implant^®^ within a D4 bone block

Once the drilling protocols were completed, implants were inserted into the bone blocks using a surgical system (Elcomed, W&H Deutschland GmbH, Bad Reichenhall, Deutschland) with a surgical contra-angle handpiece (Implant 20:1 Dentsply IH GmbH, Bensheim, Germany). Implant insertions were performed in both D4 and D2/D3 bone types (Fig. 1). Each implant system was subjected to five insertion trials for each drilling protocol and bone type to ensure consistency (Fig. 2).

### Measurement of torque

**Fig. 3:**
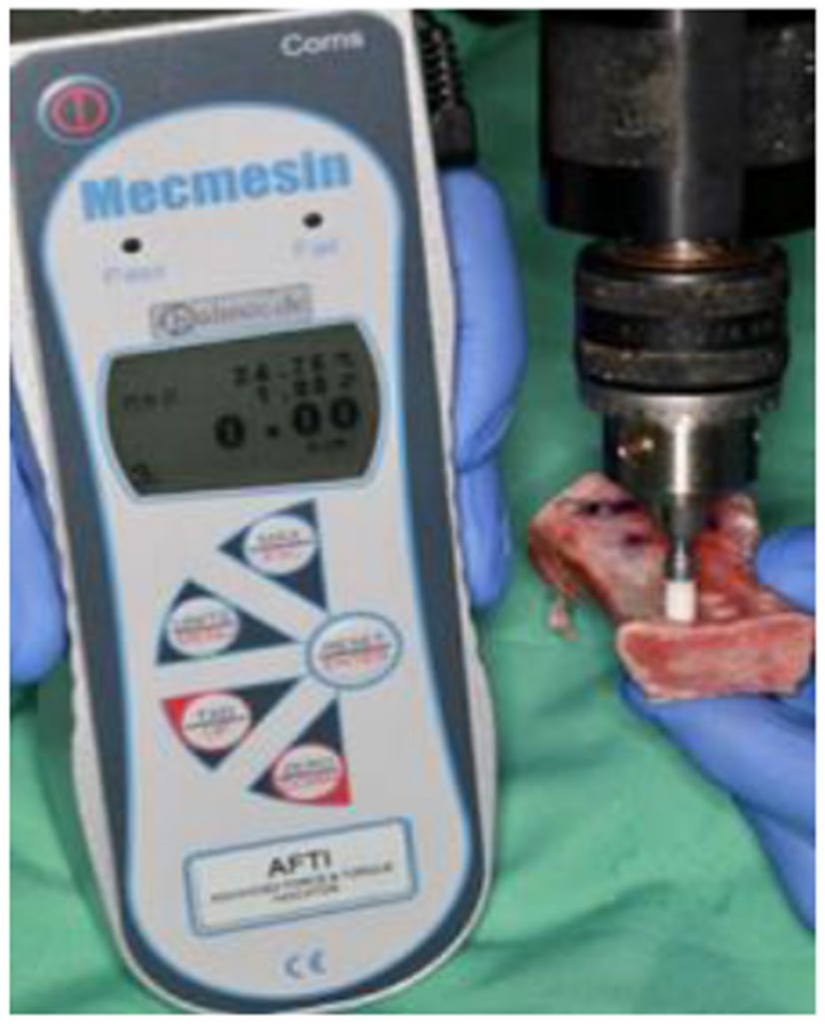
Torque measurement conducted using the AFTI force and torque indicator

The primary stability of each implant was assessed by measuring the insertion torque. A force and torque indicator (AFTI, Mecmesin PPT GmbH & Co KG, United Kingdom) was used to record the maximum torque during both the insertion and removal of each implant (Fig. 3). The maximum torque was measured during the final rotation of implant insertion and the initial rotation of implant removal.

### Statistical analysis

Statistical analysis was conducted using IBM SPSS Statistics version 23 (IBM Corporation, Armonk, New York, USA) under the supervision of the Institute of Medical Biometry, Epidemiology, and Informatics at the Mainz University Medical Centre. Non-parametric methods were employed, as the assumption of normal distribution could not be verified. Group comparisons were conducted using the Mann-Whitney U test due to the lack of normality in the data. Descriptive statistics, including medians and interquartile ranges, were calculated, and the measurement results were visually represented using boxplots. The confidence level was set at 95%, and differences were considered statistically significant at *p* < 0.05.

## Results

This study evaluated the primary stability of four commercially available zirconia implant systems, with the Ceramic.implant^®^ system tested in two different diameters (4 mm and 5 mm). The maximum insertion torque was measured across all systems using four distinct drilling protocols (B1-B4) in both D4 and D2/D3 bone types. The findings are presented below.

### PURE® ceramic implant

The PURE^®^ Ceramic Implant achieved a mean maximum insertion torque of 37.81 Ncm (SD ± 12.82) in D4 bone when using a full thread cut (B4), which fell within the manufacturer’s recommended range of 35 Ncm. A 2/3 thread cut (B3) resulted in a torque of 34.68 Ncm (SD ± 10.43), also within the acceptable range. However, with a 1/3 thread cut (B2), the torque increased to 54.15 Ncm (SD ± 15.54), while no thread cutting (B1) produced the highest torque of 56.43 Ncm (SD ± 13.33), both exceeding the recommended limit.

In D2/D3 bone, the implant system recorded a mean torque of 42.5 Ncm (SD ± 9.10) with a full thread cut (B4) and 59.81 Ncm (SD ± 10.86) with a 2/3 thread cut (B3), both exceeding the recommended range. Due to the high torque results from B3, no data were collected for B1 and B2 in this bone type. The comparison between B3 and B4 in D4 bone showed no statistically significant difference (*p* = 0.465) (Table 3).

**Table 3:** Mean insertion torques (Ncm) for different drilling protocols and their corresponding recommended torque values (Ncm)

Implant System	Protocol	Mean Torque in D4 (Ncm ± SD)	Mean Torque in D2/D3(Ncm ± SD)	Recommended Torque (Ncm)
PURE^®^ Ceramic Implant	B1	56.43 ± 13.33	-	35
	B2	54.15 ± 15.54	-	35
	B3	34.68 ± 10.43	59.81 ± 10.86	35
	B4	37.81 ± 12.82	42.50 ± 9.10	35
Ceramic.implant^®^ (⌀ 4 mm)	B1	36.79 ± 5.67	-	25–35
	B2	39.40 ± 11.57	-	25–35
	B3	33.55 ± 8.46†	37.90 ± 4.96	25–35
	B4	16.37 ± 5.83	22.69 ± 5.54	25–35
Ceramic.implant^®^ (⌀ 5 mm)	B1	43.02 ± 10.99	-	25–35
	B2	43.30 ± 12.70	-	25–35
	B3	25.35 ± 6.32†	82.25 ± 13.53	25–35
	B4	15.22 ± 4.97	24.68 ± 7.06	25–35
Zeramex XT^®^	B1	50.09 ± 5.75†	-	20–45
	B2	44.43 ± 11.35†¥	-	20–45
	B3	29.26 ± 14.88†	57.42 ± 6.72	20–45
	B4	15.78 ± 5.77	27.66 ± 4.82	20–45
Ceralog Monobloc^®^ M10	B1	32.15 ± 9.88	-	35
	B2	35.83 ± 7.70	-	35
	B3	27.32 ± 8.78	52.12 ± 5.14	35
	B4	19.10 1.87	32.15 ± 3.56	35

Displays the mean insertion torques (Ncm) with standard deviations (SD) for four zirconia implant systems across different drilling protocols in D4 and D2/D3 bone qualities. Comparisons should be made within the same implant system (i.e., between protocols B1 to B4 for each implant system), and not across different implant systems. (Ncm; Newton centimeters, B1-B4; drilling protocols B1 to B4, D2/D3/D4; bone quality types)

**†**: Indicates a significant difference in torque in D4 bone compared to B4 within the same implant system (*p* < 0.05)

¥: Indicates a significant difference in torque in D4 bone compared to B3 within the same implant system

-: No data for B1 and B2 were collected, as the values exceeded the manufacturer’s recommended range

### Ceramic.implant® (4 mm diameter)

For the Ceramic.implant^®^ with a 4 mm diameter, insertion torque in D4 bone reached 16.37 Ncm (SD ± 5.83) with a full thread cut (B4), which was below the manufacturer’s recommended range of 25–35 Ncm. Increasing the thread cut to 2/3 (B3) raised the torque to 33.55 Ncm (SD ± 8.46), falling within the recommended range. A further increase to 39.40 Ncm (SD ± 11.57) was observed with a 1/3 thread cut (B2), and no thread cutting (B1) resulted in a torque of 36.79 Ncm (SD ± 5.67), both exceeding the maximum recommended torque.

In D2/D3 bone, the system produced mean torques of 22.69 Ncm (SD ± 5.54) with a full thread cut (B4) and 37.90 Ncm (SD ± 4.96) with a 2/3 thread cut (B3), with B3 exceeding the recommended 35 Ncm. B1 and B2 were not tested, as B3 results already surpassed the manufacturer’s maximum limit. The difference between B3 and B4 in D4 bone was statistically significant (*p* = 0.009) (Table 3).

### Ceramic.implant® (5 mm diameter)

For the Ceramic.implant^®^ with a 5 mm diameter, the mean maximum insertion torque in D4 bone was 15.22 Ncm (SD ± 4.97) with a full thread cut (B4), falling below the manufacturer’s recommended range of 25–35 Ncm. A 2/3 thread cut (B3) increased the torque to 25.35 Ncm (SD ± 6.32), within the acceptable range. However, with a 1/3 thread cut (B2), the torque rose to 43.30 Ncm (SD ± 12.70), and without thread cutting (B1), a similar torque of 43.02 Ncm (SD ± 10.99) was recorded, both exceeding the recommended maximum.

In D2/D3 bone, the implant system displayed torques of 24.68 Ncm (SD ± 7.06) and 82.25 Ncm (SD ± 13.53) with full and 2/3 thread cuts (B4 and B3), respectively. The latter exceeded the manufacturer’s recommended range, so no data were collected for B1 and B2. The difference between B3 and B4 in D4 bone was statistically significant (*p* = 0.028) (Table 3).

### Zeramex XT®

With the Zeramex XT^®^ system, insertion torque in D4 bone reached 15.78 Ncm (SD ± 5.77) with a full thread cut (B4), which fell below the manufacturer’s recommended range of 20–45 Ncm. Increasing the thread cut to 2/3 (B3) resulted in a torque of 29.26 Ncm (SD ± 14.88), still within the acceptable range. However, the system reached 44.43 Ncm (SD ± 11.35) with a 1/3 thread cut (B2), and no thread cutting (B1) produced a torque of 50.09 Ncm (SD ± 5.75), which exceeded the recommended range.

In D2/D3 bone, the system produced torques of 27.66 Ncm (SD ± 4.82) and 57.42 Ncm (SD ± 6.72) with full thread (B4) and 2/3 thread cuts (B3), respectively, with B3 exceeding the recommended maximum of 45 Ncm. Data for B1 and B2 were not collected. Statistically significant differences were observed between B3 and B4 in D4 bone (*p* = 0.028), as well as between B2 and B4 (*p* = 0.009) and B2 and B3 (*p* = 0.047) (Table 3).

### Ceralog Monobloc® M10

The Ceralog Monobloc^®^ M10 system demonstrated a mean maximum insertion torque of 19.10 Ncm (SD ± 1.87) in D4 bone with a full thread cut (B4), which is below the manufacturer’s recommended maximum of 35 Ncm. Increasing the thread cut to 2/3 (B3) led to a torque of 27.32 Ncm (SD ± 8.78), while a 1/3 thread cut (B2) resulted in a torque of 35.83 Ncm (SD ± 7.70), exceeding the manufacturer’s recommended limit. No thread cutting (B1) produced a mean torque of 32.15 Ncm (SD ± 9.88), within the recommended range.

In D2/D3 bone, the system produced mean torques of 32.15 Ncm (SD ± 3.56) with a full thread cut (B4) and 52.12 Ncm (SD ± 5.14) with a 2/3 thread cut (B3), with B3 exceeding the manufacturer’s recommended limit. No statistically significant difference was observed between B3 and B4 in D4 bone (*p* = 0.117), although a trend toward significance was noted. Similarly, no significant difference was found between B1 and B3 (*p* = 0.465) (Table 3).

## Discussion

This study aimed to evaluate the effect of varying drilling protocols on the primary stability of zirconia implants in different bone types, particularly in soft (D4) bone, where achieving adequate primary stability is challenging. The null hypothesis, which proposed that no improvement in primary stability would occur with a reduced thread cut, was rejected. The data clearly demonstrated that reducing the thread cut in the drilling protocols (as in protocols B1 and B2) resulted in a significant increase in insertion torque, leading to enhanced implant stability in both D4 and D2/D3 bone.

The findings of this study may be attributed to several factors, including bone quality, surface roughness and macrogeometry. Across all implant systems, primary stability, as measured by insertion torque, was greater in denser D2/D3 bone compared to softer D4 bone. However, specific variations were observed between the implant systems, emphasizing the importance of implant selection based on clinical conditions.

Bone quality played a pivotal role in the effects of the drilling protocols. In D4 bone, where primary stability is more difficult to achieve, reduced thread cutting led to substantial improvements in insertion torque. In D2/D3 bone, the changes were less dramatic, though stability still improved. This variation suggests that clinicians should adjust their drilling techniques to the bone quality at the implant site, as a one-size-fits-all approach may lead to suboptimal outcomes. The findings have important implications for immediate loading protocols, where high primary stability is essential. In D4 bone, the increased stability from reduced thread cutting may make immediate loading more feasible. Zarrabi et al. [13] found that there was no significant difference in survival rates between immediately loaded and traditionally healed implants when a high initial stability of around 50 Ncm was maintained. Immediate loading protocols, therefore, rely heavily on the surgeon’s ability to adapt the drilling protocol to the specific bone quality, as per manufacturers’ recommendations [14]. However, an increase in primary stability does not apply universally across all systems. Each implant system has a maximum recommended insertion torque that should not be exceeded to avoid implant damage. Exceeding the manufacturer’s recommended maximum insertion torque may result in thread chipping, damage to the implant neck caused by the insertion tool, and, in some cases, complete implant fracture during the insertion procedure [12]. As demonstrated in this study, if sufficient primary stability is achieved without altering the protocol and approaching the maximum recommended torque, any further increase in torque may be detrimental. For instance, the PURE^®^ Ceramic Implant achieved a mean insertion torque of 37.81 Ncm (SD ± 12.82) with a full thread cut in D4 bone, closely aligning with the manufacturer’s recommendation of 35 Ncm. This indicates that no modification to the drilling protocol is needed in D4 bone for optimal primary stability with this implant system. On the other hand, the Zeramex^®^ XT, Ceralog Monobloc^®^ M10 and Ceramic.implant^®^ exceeded the maximum recommended torque limits in D4 bone when subjected to B1 and B2 drilling protocols which introduces potential risks for mechanical failure. These findings align with Gahlert et al. [11], who advise against excessive insertion torque, particularly in softer bone, where the risk of implant fracture is higher.

In the denser D2/D3 bone, all implant systems exceeded the maximum recommended torques when the thread cut was reduced, highlighting the ability of each system to achieve primary stability suitable for immediate loading (≥ 20 Ncm − 45 Ncm). These findings are compatible with Arosio et al. [10] who stated that, reducing the thread cut or underpreparing the implant bed may be crucial in cases involving softer bone types than D4, particularly when bone augmentations are performed to compensate for bone loss.

Variability in response across different implant systems highlights the role of implant design in achieving optimal primary stability [9]. While all systems showed increased stability with reduced thread cuts, some were more likely to exceed safe torque limits. This suggests that implant design, particularly thread geometry and material properties, significantly influences the outcome of drilling protocol modifications.

The differences observed in primary stability among the implant systems tested may be attributed, in part, to variations in their macrogeometry. Although thread pitch and geometry were not directly measured in this study, prior research suggests that factors such as flank diameter and thread type could influence stability outcomes [9]. Studies have shown that larger flank diameters are generally associated with higher primary stability, particularly in both D4 and D2/D3 bone types [3, 15]. The PURE^®^ Ceramic Implant, with its pointed thread and relatively larger flank diameter, may offer enhanced stability compared to the Ceramic.implant^®^ and Ceralog Monobloc^®^ M10, which have less pronounced geometries. Similarly, the Zeramex^®^ XT’s pointed thread design may contribute to its stability, potentially similar to the PURE^®^ Ceramic Implant. The asymmetrical saw thread of the Ceramic.implant^®^ could provide better occlusal force transmission and partial bone compaction, which might reduce healing time. On the other hand, the Ceralog Monobloc^®^ M10’s blunt thread and narrower flank cylinder may reduce its potential for mechanical anchorage, while offering greater resistance to thread chipping compared to more aggressive designs.

The variation in maximum insertion torque among different implant systems using the same drilling protocol and bone quality may be partly due to differences in surface roughness. In this study, the surface roughness of the implants decreased in the following order: Straumann PURE^®^ Ceramic Implant (ZLA^®^ surface) at 1.27 μm (SD ± 0.24), Ceralog Monobloc^®^ M10 at 1.22 μm (SD ± 0.36), Ceramic.implant^®^ (cer.face^®^ surface) at 1.05 μm (SD ± 0.17), and Zeramex^®^ XT (ZERAFIL™ surface) at 0.95 μm (SD ± 0.77) [16]. This trend was mirrored in the maximum insertion torque in D4 bone, where the PURE^®^ Ceramic Implant had the highest torque at 37.81 Ncm (SD ± 12.82), followed by the Ceralog Monobloc^®^ M10 at 19.10 Ncm (SD ± 1.87), the Ceramic.implant^®^ at 16.37 Ncm (SD ± 5.83), and the Zeramex^®^ XT at 15.78 Ncm (SD ± 5.77).

In D2/D3 bone, a similar pattern was observed. The PURE^®^ Ceramic Implant had the highest torque at 42.5 Ncm (SD ± 9.10), followed by the Ceralog Monobloc^®^ M10 at 32.15 Ncm (SD ± 3.56), the Zeramex^®^ XT at 27.66 Ncm (SD ± 4.82), and the Ceramic.implant^®^ at 22.69 Ncm (SD ± 5.54). While surface roughness likely plays a role in these results, other factors such as implant geometry and the specific drilling protocol may also influence primary stability.

Previous studies support the link between surface roughness and insertion torque. Dos Santos et al. [17] found that increased roughness correlates with higher friction and insertion torque. Similarly, Tabassum et al. [18] demonstrated that roughness and undersized implant bed preparation enhance primary stability in artificial bone up to a cortical thickness of 2 mm, although neither factor significantly affects stability at greater thicknesses.

**Fig. 4:**
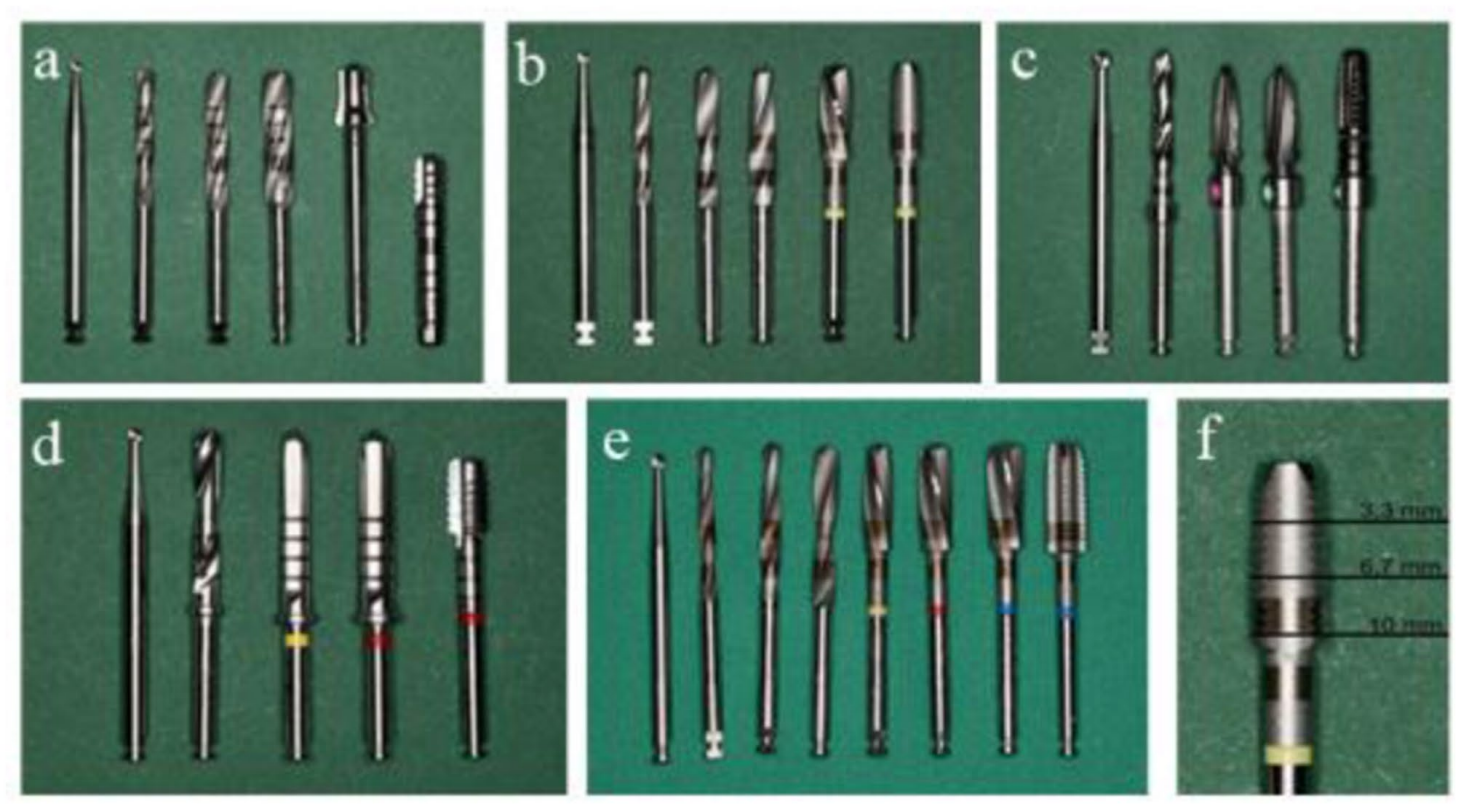
Drill configurations for each implant system: (**a**) PURE^®^ Ceramic Implant, (**b**) Ceramic.implant^®^ 4 × 10 mm, (**c**) Zeramex^®^ XT, (**d**) Ceralog Monobloc^®^, and (**e**) Ceramic.implant^®^ 5 × 10 mm. (**f**) Thread cutting depths indicated on the drill at 3.3 mm, 6.7 mm, and 10 mm

The method of implant bed preparation may also influence primary stability, even when implant design and bone quality remain constant. As demonstrated in Fig. 4, the drills provided by different manufacturers vary significantly in terms of diameter, thread pitch, chip space, and sharpness, which impacts both thermal and mechanical stress on the bone [19]. Although this study does not aim to examine the effects of drilling protocols on bone compaction or implant site preparation, it is evident that the protocol used must align with the implant design. Since no universal standard exists for drill design, differences between manufacturers in drill geometry, bone compaction, or thread cutting may influence insertion torque [7].

Increasing the diameter of an implant is generally considered to enhance primary stability by increasing the surface area available for bone contact. However, in this study, the results didn’t follow this expectation. When comparing the 4 mm and 5 mm Ceramic.implant^®^ systems in soft D4 bone, the smaller 4 mm implant actually showed a higher insertion torque (33.55 Ncm) than the larger 5 mm implant (25.35 Ncm). This outcome was surprising, as the larger implant should theoretically be more stable. There are a few possible reasons for this unexpected result. Differences in how consistently the implant bed was prepared, variations in the quality of the bone, or even measurement errors could have influenced the results. These factors may have caused the 4 mm implant to appear more stable in this case. Despite these unexpected findings, both implant sizes showed improved stability when a different drilling technique (B3) was used. The statistical analysis confirmed that this improvement was significant for both implants, with p-values of 0.009 and 0.028. This supports the hypothesis that changing the drilling protocol can enhance primary stability, even if the results don’t always follow theoretical expectations [5].

The study offers valuable insights but has several limitations. First, the use of ex vivo porcine bone, while a common substitute for human bone, cannot fully replicate the variability of in vivo human conditions. Despite efforts to ensure homogeneity, natural irregularities in bone, such as marrow-like or cartilage regions, could have affected the insertion torque measurements, particularly in softer D4 bone. The lack of three dimensional imaging also limited the ability to assess these variations. Additionally, the bone blocks were not perfectly cuboidal, and varying cortical thickness may have provided more stability at some implant sites than typically seen in human cancellous jawbone. The surgical technique, while mimicking clinical conditions, lacked a drilling template, which could have introduced slight deviations in implant sites, despite being performed by the same surgeon.

## Conclusions

Within the limitations of this study, it can be concluded that reducing the thread cut enhances primary stability, as indicated by insertion torque. However, this adjustment may not always be necessary, depending on the implant’s geometry and bone quality. In softer bone (D4), certain zirconia implants achieved optimal stability without thread reduction due to their design. These findings emphasize the importance of understanding the behavior of each zirconia implant system in relation to primary stability. Drilling protocols should be adjusted to the specific implant system and bone characteristics to optimize clinical outcomes.

## Data Availability

No datasets were generated or analysed during the current study.

## Abbreviations

D1: Dense cortical bone
D2: Dense-to-moderate cortical bone with thick trabecular bone
D3: Thin cortical bone with fine trabecular bone
D4: Very soft, primarily trabecular bone
B1-B4: Drilling protocols 1–4
ZLA: Zirconia Large-grit Acid-etched
ATZ: Aluminia-thoughened-Zirconia
HIP: Hot Isostatic Postcompaction
CIM: Ceramic Injection Moulding
ZrO_2_: Zirconium dioxide
SD: Standard deviation
Ncm: Newton centimeters
